# Catastrophic health expenditure and distress financing of breast cancer treatment in India: evidence from a longitudinal cohort study

**DOI:** 10.1186/s12939-024-02215-2

**Published:** 2024-07-23

**Authors:** Sanjay K. Mohanty, Tabassum Wadasadawala, Soumendu Sen, Suraj Maiti, Jishna E

**Affiliations:** 1 Department of Population and Development, International Institute for Population Sciences, Mumbai, 400 088 India; 2 Department of Radiation Oncology, Advanced Centre for Treatment, Research and Education in Cancer (ACTREC), Tata Memorial Centre, Homi Bhabha National Institute, Navi Mumbai, 410 210 India; 3 International Institute for Population Sciences, Mumbai, 400 088 India

**Keywords:** Breast cancer, Catastrophic health expenditure, Distress financing, Mumbai, India

## Abstract

**Objective:**

To estimate the catastrophic health expenditure and distress financing of breast cancer treatment in India.

**Methods:**

The unit data from a longitudinal survey that followed 500 breast cancer patients treated at Tata Memorial Centre (TMC), Mumbai from June 2019 to March 2022 were used. The catastrophic health expenditure (CHE) was estimated using households’ capacity to pay and distress financing as selling assets or borrowing loans to meet cost of treatment. Bivariate and logistic regression models were used for analysis.

**Findings:**

The CHE of breast cancer was estimated at 84.2% (95% CI: 80.8,87.9%) and distress financing at 72.4% (95% CI: 67.8,76.6%). Higher prevalence of CHE and distress financing was found among rural, poor, agriculture dependent households and among patients from outside of Maharashtra. About 75% of breast cancer patients had some form of reimbursement but it reduced the incidence of catastrophic health expenditure by only 14%. Nearly 80% of the patients utilised multiple financing sources to meet the cost of treatment. The significant predictors of distress financing were catastrophic health expenditure, type of patient, educational attainment, main income source, health insurance, and state of residence.

**Conclusion:**

In India, the CHE and distress financing of breast cancer treatment is very high. Most of the patients who had CHE were more likely to incur distress financing. Inclusion of direct non-medical cost such as accommodation, food and travel of patients and accompanying person in the ambit of reimbursement of breast cancer treatment can reduce the CHE. We suggest that city specific cancer care centre need to be strengthened under the aegis of PM-JAY to cater quality cancer care in their own states of residence.

**Trial Registration:**

CTRI/2019/07/020142 on 10/07/2019.

**Supplementary Information:**

The online version contains supplementary material available at 10.1186/s12939-024-02215-2.

## Introduction

Increasing incidence of breast cancer and catastrophic health expenditure (CHE) are concomitant worldwide. In low-and-middle income countries (LMICs), early onset of cancer coupled with low screening and late diagnosis leads to high mortality and treatment complications [1, 2]. In 2023, of the 2.3 million breast cancer cases diagnosed worldwide, about 0.69 million succumbed to the disease [3]. Despite increase in health insurance coverage, the catastrophic health expenditure has been increasing in many countries [4]. Disease specific estimates suggest highest financial catastrophe of cancer treatment within and between the countries [5, 6]. Understanding the need, the UN had recommended CHE and impoverishment as two of the indicators to monitor sustainable development goal (SDG) 3.8 [4]. 

In LMICs, the household economic burden of breast cancer is profound owing to high mortality, disability, cost and out-of-pocket (OOP) payment [7–9] High cost of treatment increases the chance on untreated morbidity by limiting the access to healthcare which may result in high mortality and lower quality of life [10, 11]. The financial protection mechanism for cancer is inadequate and varies largely by geography, demography, and wealth [12–14]. 

Literature suggests that countries with higher Human Development Index (HDI) incurred lower CHE (23.4%) for cancer treatment compared to countries with lower HDI (67.9%) [15]. The CHE of end-of-life cancer patients in China was estimated at 94.3% in urban areas and 96.1% in rural areas [16]. The CHE of breast cancer patients at the 30%, 40% and 50% threshold was estimated at 46%, 43% and 32% respectively [17]. The overall incidence of CHE in China was 88% and 66% before and after insurance compensation, respectively [18]. In Malaysia, the CHE of colorectal cancer was 48% and that of oral cancer was 87% [19]. In India, 62% of cancer patients incurred CHE and 30% had distress financing with a higher incidence among poor and treated in private hospital [20, 21]. A cohort study from five tertiary hospitals in India estimated CHE at 90.1% for colorectal cancer [22]. Employment status, distance and insurance coverage are the major determinants contributing to escalation of CHE [22]. The probability of incurring CHE was also high for longer duration of treatment, type of health insurance, poor households, and household size [17, 19, 23, 24]. 

India, has the third highest breast cancer cases worldwide; accounting 8.4% of 2.3 million breast cancer cases [25]. However, the estimates are at lower side owing to low screening (only 0.9% of women were ever screened) for breast cancer [2]. The breast cancer treatment is also very limited in the country, largely located in city centres and expensive. On the other hand, the health spending in the country is largely met by out-of-pocket payment and household is the main source of financing. Though studies estimated the cost, OOP and catastrophic health spending of cancer care, there is paucity of studies on estimation of CHE and distress financing of breast cancer in India. Thus, we have conceptualized the study with the following rationale. First, breast cancer is the single largest cause of cancer in India and has been increasing with time. Women in India are often neglected in terms of healthcare and have low socio-economic conditions within the households. Second, we came across three small scale studies that estimated the CHE of cancer but not by specific type of cancer [6, 21, 26]. Third, existing studies used cross sectional data at a point in time that did not adequately capture the cost of cancer treatment as cancer treatment continues over a period and through multiple providers. Recall bias in reporting of treatment cost is likely to be high in cross sectional surveys and there is no study based on longitudinal data. We have followed a longitudinal design that collected expenditure data. Fourth, evidence suggests increase in health insurance coverage in the country; from 4.9% in 2005-06 to 41% by 2019-21 [27–29]. However, it is not known how much health insurance reduces the CHE of breast cancer patients. In context of above rationale, the objective of this paper is to estimate the incidence of CHE, distress financing and impoverishment of breast cancer treatment in India. We also examined the effect of reimbursement on the reduction of CHE of breast cancer households in India.

## Data & methods

A total of 500 breast cancer patients were followed over a period of 34 months (June, 2019—March, 2022) in a tertiary cancer hospital (Tata Memorial Centre (TMC), Mumbai, India). The study was a collaboration between TMC, Mumbai and the International Institute for Population Sciences (IIPS), Mumbai [30]. The participation in the survey was voluntary. A written consent was obtained from the study participants as well as their accompanying persons before starting of the survey. No compensation was paid to the participants. Data were collected by trained medical social workers from breast cancer patients and accompanying persons at the TMC. The medical social workers (MSWs) were trained by the principal investigators of the project before starting the survey. Also, MSWs were guided by the senior project officer and research scholar associated with this project. Frequent monitoring visit was conducted to ensure the data quality. Data validation was carried out using electronic medical records of the hospital for selected components and missing information were monitored on weekly basis. The survey was conducted within the hospital premise at TMC where patients were provided services.

A total of 500 patients were included (twice higher than the required estimates) for segregated analyses [31]. The sampling frame and the profile of selected sample have been described elsewhere [32]. Data were collected using household and individual schedules at the time of registration (termed as baseline) and at the time of completion of treatment (termed as endline). Information on direct medical and non-medical costs of treatment was collected at each visit of patients to the hospital which is unique feature of the study. The cost of registration, admission, investigation, medicine, surgery, systemic therapy and radiotherapy were classified under direct medical cost. Similarly, expenditure on, food, accommodation, and travel were classified as direct non-medical cost. Detail information of coping mechanisms for cancer treatment from multiple sources such as income, savings, selling of assets, borrowing, loans and insurance was collected at the endline and used in estimating distress financing. Our estimated cost is only for treatment at TMC and did not include the cost incurred before coming to TMC. An abridged version of consumption schedule (16 items) was canvassed to estimate the consumption expenditure (SM Table 1). Data on consumption includes consumption of food items, expenditure on utility bills, travel, entertainment, habits, consumer services, rentals (house) in a reference period of 30 days and usual expenditure on education, clothes, insurance premium (life, health) and other expenditure (if any) in a reference period of last one year. The consumption expenditure was standardized to 30 days to derive household consumption expenditure. The monthly per capita consumption expenditure (MPCE) was derived by dividing the household consumption expenditure by the household size. The consumption of the household was collected at base line (time of first interview) and endline (after completion of treatment) for patients and accompanying person as well as the other members of the households.

**Table 1:** Descriptive statistics of household expenditure, income source and reimbursement for breast cancer patients’ households seeking treatment at TMC, Mumbai

Variables	(Mean, Median, %)	95% CI, IQR
Mean number of visits to the hospital	50	[48, 51]
Mean age at diagnosis (years)	46.5	(45.5, 47.5)
Median MPCE (in INR)	3953	[2576, 6152]
Median OOP payment (in INR)	126,988	[54,517, 248,612]
Mean OOP payment (in INR)	186,461	[167,666, 205,257]
Monthly mean OOP payment (in INR)	20,419	[18,468, 22,370]
Percentage reimbursed	74.4	NA
Mean amount reimbursed (in INR)	71,724	[61,747, 81,701]
Reimbursement as a share of treatment cost at TMC	30.2	NA
Percentage of rural patients	54.3	NA
Percentage households with agriculture as main income source	12.6	NA
Percentage households with labour as main income source	24.0	NA
Percentage of patients with all three treatment modalities (radiotherapy, systemic therapy and surgery)	83.2	NA
N	429	NA

*NA: Not Applicable. Confidence interval are not shown for variables which are sample characteristic and not estimates*

### Outcome variable

The incidence and intensity of catastrophic health expenditure, impoverishment and distress financing were the primary outcome variables in the study. The treatment cost at TMC for direct medical and non-medical cost was collected for each subsequent visit to TMC, and was aggregated for the total number of visits made to TMC. The OOP payment is defined as treatment cost at TMC less of reimbursement. The aggregate OOP payment during treatment was divided by duration of treatment (days) and then multiple by 30 to derive monthly OOP payment. The CHE was derived from monthly OOP payment and consumption expenditure. A patient was said to incur distress financing if the cancer treatment was met either by any of the following means; selling assets jewellery, property or by taking loans.

### Statistical analysis

The CHE was estimated for the patients who completed treatment at TMC. The CHE was calculated using the capacity to pay approach recommended by WHO [33, 34]. We used consumption expenditure $\left({\text{C}}_{\text{i}}\right)$ excluding health expenditure and median food expenditure in deriving subsistence expenditure (${\text{S}\text{E}}_{\text{i}}$). A household incurred CHE (1 = Yes, 0 = No) if

1 $${E_i} = \frac{{OO{P_i}}}{{{C_i} - S{E_i}}} \ge 40\% ,\,CHE = 1,\,0\,{\rm{otherwise}}.$$

E_i_ is an indicator showing whether household incurred CHE or not.

OOPi is the out-of-payment of breast cancer treatment of ith household

2 $${\rm{Incidence}}\,{\rm{of}}\,{\rm{CHE}}\,{\rm{ = }}\,\frac{{\rm{1}}}{{\rm{N}}}\sum\nolimits_{{\rm{i = 1}}}^{\rm{N}} {{{\rm{E}}_{\rm{i}}}}$$

where N is the total number of households.

The intensity of CHE was calculated as follows:

3 $${\rm{Incidence}}\,{\rm{of}}\,{\rm{CHE}}\,{\rm{ = }}\,\frac{{\rm{1}}}{{\rm{N}}}\sum\nolimits_{{\rm{i = 1}}}^{\rm{N}} {\left( {\frac{{{\rm{OO}}{{\rm{P}}_{\rm{i}}}}}{{{\rm{CTP}}}}{\rm{ - 0}}{\rm{.4}}} \right){{\rm{E}}_{\rm{i}}}}$$

A non-poor household was said to be impoverished (IMPO) by OOP payments if their consumption expenditure fell short of subsistence expenditure following health expenditure [29], i.e.

4 $$IMP{O_i}:{C_i} > S{E_i}\,and\,{C_i} - OO{P_i} < S{E_i}$$

A set of logistic regression models were used to determine the significant predictors of distress financing. In the first step (Model 1), the association of CHE with distress financing was estimated. In the second step (Model 2), the patients’ characteristics such as age, type of patient, marital status, stage of cancer, and in full model, the household characteristics were included. All analyses were performed in Stata version 17.0.

## Results

**Fig. 1:**
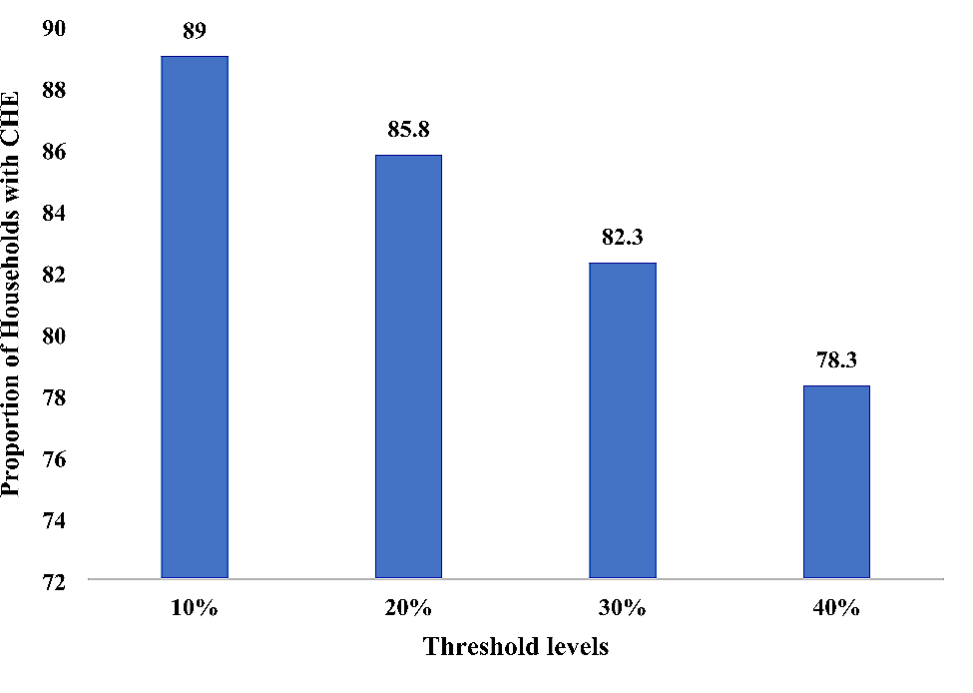
OOP payment as a share of consumption expenditure at varying thresholds among breast cancer households

The median MPCE, excluding health expenditure, was ₹3,953 (95% CI: 3604, 4230). The median and mean OOP payment was ₹126,988 (95% CI: 109749, 144437) and ₹186,461 (95% CI: 167666, 205257) respectively (Table 1). Treatment modalities included surgery, systemic therapy and radiotherapy. About 83% patients had received all the three types of treatment modalities, surgery, systemic therapy and radiotherapy. Though majority of the breast cancer patients had some type of reimbursement, it covered only 30.2% of the total treatment expenses. The mean amount reimbursed was ₹78,016 (95% CI: 66,291, 89,741). About 24% of the households in the sample were labourer households and 12.6% depended on agriculture as primary income source. Almost half of the patients resided in rural areas. The OOP payment as a share of consumption expenditure at the threshold was at 89.0% at the 10% threshold, and 78.3% at the 40% threshold (Fig. 1).

Table 2 presents the estimates of incidence, intensity of CHE, impoverishment and distress financing by socio-economic and demographic characteristics. The socio-economic gradient for CHE and impoverishment is strong. About 84.6% of the households incurred CHE, while 55.0% of the households were impoverished during treatment. The intensity of CHE and impoverishment declined across each MPCE quintile. Patients from rural areas who had no/ low educational attainment incurred higher CHE and distress financing as compared to urban and higher educated patients. The households with general or non-chargeable patients had higher CHE compared to households of private patients but the latter had lower prevalence of impoverishment than the former. Breast cancer patients who came from the states of Bihar and Uttar Pradesh had higher CHE and impoverishment in comparison to patients from Maharashtra. On an average, households incurred CHE two times more than their capacity to pay. The extent of distress financing was higher among younger women, poor, rural, uneducated, labourer households, general patients, those with longer treatment duration and those diagnosed in advanced stage of breast cancer. Households of patients receiving all three treatment modalities were two times more impoverished than households of patients receiving partial of the treatment modalities.

**Table 2:** Incidence, intensity of CHE, impoverishment, and distress financing by socio-demographic and economic characteristics among breast cancer patients

Variables		Incidence of CHE		Intensity of CHE	Impoverishment		Distress Financing	
	**n**	**%**	**95% CI**	**Mean**	**%**	**95% CI**	%	**95% CI**
**Age**								
Up to 45	202	85.2	[79.5, 89.8]	1.2	53.5	[46.3, 60.5]	74.0	[68.0, 80.4]
Over 45 years	227	84.1	[78.7. 88.6]	2.8	56.4	[49.7, 62.9]	71.0	[64.3, 76.6]
**Marital Status**								
Other	63	84.1	[72.7, 92.1]	1.2	41.3	[29.0, 54.4]	66.0	[52.3, 77.3]
Currently Married	366	84.7	[80.6, 88.2]	2.2	57.4	[52.1, 62.5]	74.0	[68.6, 78.0]
**MPCE quintile**								
Poorest	83	84.3	[74.7, 91.4]	7.3	63.9	[52.6, 74.1]	73.8	[62.7, 82.3]
Poorer	78	83.3	[73.2, 90.8]	1.3	56.4	[44.7, 67.6]	79.5	[68.8, 87.8]
Middle	89	84.3	[75.1, 91.1]	1.0	55.1	[44.1, 65.6]	73.6	[63.0, 82.4]
Richer	89	85.4	[76.3, 92.0]	0.8	48.3	[37.6, 59.2]	64.7	[53.6, 74.8]
Richest	90	85.6	[76.6, 92.1]	0.6	52.2	[41.4, 62.9]	70.9	[60.1, 80.2]
**Place of residence**								
Urban	196	78.1	[71.6, 83.6]	1.8	43.9	[36.8, 51.1]	69.0	[61.8, 75.4]
Rural	233	90.1	[75.6, 93.6]	2.2	64.4	[57.9, 70.5]	75.0	[69.1. 80.7]
**Household Size**								
1–4 members	217	82.5	[76.8, 87.3]	2.1	53.9	[47.0, 60.7]	69.1	[62.5, 75.2]
5 or more members	212	86.8	[81.5, 91.0]	1.9	56.1	[49.2, 62.9]	71.2	[64.6, 77.2]
**Level of Education**								
Never Attended	99	89.9	[82.2, 95.0]	1.4	60.6	[50.3, 70.3]	79.0	[69.4, 86.4]
Primary	36	83.3	[67.2, 93.7]	5.7	55.6	[38.1, 72.1]	72.0	[54.8, 85.8]
Secondary	167	82.0	[75.4, 87.5]	2.3	47.9	[40.1, 55.8]	74.0	[66.4, 80.5]
Higher Secondary	50	90.0	[78.2, 96.7]	1.1	62.0	[47.2, 75.3]	79.0	[65.0, 89.5]
Above HS	77	80.5	[69.9,88.7]	1.1	58.4	[46.7, 69.6]	56.0	[43.4, 67.3]
**Religion**								
Hindu	332	85.2	[81.0, 88.9]	2.2	55.7	[50.2, 61.1]	70.0	[64.1. 74.5]
Muslim	80	82.5	[72.4, 90.1]	1.3	56.3	[44.7, 67.3]	85.0	[74.7, 91.8]
Other	17	82.4	[56.6, 96.2]	1.5	35.3	[14.2, 61.7]	71.0	[44.0, 89.7]
**Caste**								
General	226	84.1	[78.6, 88.6]	1.8	55.8	[49.0, 62.3]	73.0	[66.0, 78.3]
OBC	145	86.9	[80.3, 91.9]	2.7	53.1	[44.7, 61.4]	72.0	[64.2, 79.5]
SC/ST/Other	58	81.0	[68.6, 90.1]	1.2	56.9	[43.2, 69.8]	72.0	[58.5, 83.0]
**Occupation**								
Agriculture	54	98.1	[90.1, 99.9]	4.5	66.7	[52.5, 78.9]	75.0	[61.1, 86.0]
Labour	103	86.4	[78.2, 92.4]	1.3	56.3	[46.2, 66.1]	86.0	[77.8, 92.2]
Self-employed	66	80.3	[68.7, 89.1]	1.2	57.6	[44.8, 69.7]	81.0	[69.1, 89.8]
Service	206	81.6	[75.6, 86.6]	2.0	50.5	[43.5, 57.5]	62.0	[54.9, 68.8]
**Type of patient**								
General	369	85.1	[81.0, 88.6]	2.2	54.0	[48.7, 59.1]	70.0	[68.3, 79.3]
Private	60	81.7	[69.6, 90.5]	1.0	61.7	[48.2, 73.9]	74.0	[52.1, 66.9]
**Stage of Cancer**								
Early Stage (I/II)	155	81.3	[74.2, 87.1]	1.3	52.3	[44.1, 60.3]	69.5	[71.1, 80.2]
Advanced Stage (III/IV)	274	86.5	[81.9, 90.3]	2.4	56.6	[50.5, 62.5]	74.0	[68.3, 79.2]
**Treatment type**								
At least one or two treatment modalities	72	81.9	[71.1, 90.0]	1.1	50.0	[38.0, 62.0]	65.2	[53.1, 76.1]
Systemic therapy, radiotherapy and surgery	357	85.2	[81.0, 88.7]	2.2	56.0	[50.7, 61.2]	71.1	[66.1, 75.8]
**Duration of Treatment (in months)**								
< 9 M	214	81.3	[75.4, 86.3]	2.0	53.3	[46.4, 60.1]	68.0	[41.5, 74.5]
9 M-12 M	174	87.4	[81.5, 91.9]	2.1	54.0	[46.3, 61.6]	73.0	[65.6, 79.5]
> 12 M	41	90.2	[76.9, 97.3]	1.8	68.3	[51.9, 81.9]	92.0	[78.6, 98.3]
**State**								
Maharashtra	192	75.5	[68.8, 81.4]	2.1	41.1	[34.1, 48.5]	61.0	[53.8, 68.2]
West Bengal	83	89.2	[80.4, 94.9]	1.4	71.1	[60.1, 80.5]	83.0	[72.7, 90.2]
Bihar	52	92.3	[81.5, 97.9]	4.4	59.6	[45.1, 73.0]	84.0	[71.4, 93.0]
Uttar Pradesh	40	97.5	[86.9, 99.9]	1.5	67.5	[50.8, 81.4]	86.0	[70.5, 95.3]
Other	62	91.9	[82.2, 97.3]	1.1	64.5	[51.3, 76.3]	75.0	[62.1, 85.3]
**Total**	**429**	**84.6**	**[80.8, 87.9]**	**2.0**	**55.0**	**[51.3, 59.7]**	**72.4**	**[67.8, 76.6]**

Table 3 shows the incidence and intensity of CHE before and after reimbursement. Reimbursement was any form of financial assistance, including, health insurance, employee health schemes and assistance from charitable trusts. The incidence of CHE was 98.1% before reimbursement and 84.6% after reimbursement. Thus, reimbursement reduced CHE only by 13.8% points (pp). The incidence of CHE was lower among households from Maharashtra, patients with early-stage cancer and, urban residents.

**Table 3:** Incidence and intensity of catastrophic health expenditure (CHE) before and after reimbursement among the breast cancer patients

	CHE	% Change in CHE	Intensity	% Change in Intensity
**Total**				
Before reimbursement	98.1		3.5	
After reimbursement	84.6	13.8	2.0	42.5
**Maharashtra**				
Before reimbursement	96.4		4.6	
After reimbursement	75.5	21.6	2.1	55.7
**Outside Maharashtra**				
Before reimbursement	99.6		2.7	
After reimbursement	92.0	7.6	2.0	24.8
**Early Stage**				
Before reimbursement	97.4		2.2	
After reimbursement	81.3	16.6	1.3	41.4
**Advanced Stage**				
Before reimbursement	98.6		4.3	
After reimbursement	86.5	12.3	2.4	43.2
**No comorbidity**				
Before reimbursement	98.3		2.3	
After reimbursement	85.8	12.7	1.4	40.8
**At least one comorbidity**				
Before reimbursement	97.7		6.3	
After reimbursement	82.0	16.2	3.5	44.3
**Urban**				
Before reimbursement	97.5		4.2	
After reimbursement	78.1	19.9	1.8	57.9
**Rural**				
Before reimbursement	98.7		3.0	
After reimbursement	90.1	8.7	2.2	24.4
**Poorest**				
Before reimbursement	100.0		12.8	
After reimbursement	84.4	15.6	7.3	43.4
**Richest**				
Before reimbursement	95.6		1.0	
After reimbursement	85.6	10.5	0.6	34.0
**Up to 45 years**				
Before reimbursement	98.0		2.0	
After reimbursement	85.2	13.1	1.2	42.9
**Above 45 years**				
Before reimbursement	98.2		4.9	
After reimbursement	84.1	14.4	2.8	42.5
**1–4 members in household**				
Before reimbursement	98.2		4.2	
After reimbursement	82.5	16.0	2.1	51.1
**5 or more members in household**				
Before reimbursement	98.1		2.8	
After reimbursement	86.8	11.5	2.0	30.0
**General Patients**				
Before reimbursement	98.4		3.8	
After reimbursement	85.1	13.5	2.2	42.7
**Private Patients**				
Before reimbursement	96.7		1.67	
After reimbursement	84.1	13.0	0.99	40.7
**All the treatment modalities (systemic therapy, radiotherapy and surgery)**				
Before reimbursement	98.6		3.9	
After reimbursement	85.2	13.6	2.2	42.8
**At least one or two of the treatment modalities**				
Before reimbursement	95.8		1.9	
After reimbursement	81.9	14.5	1.1	42.4

Table 4 depicts sources of financing the breast cancer treatment. Among all the breast cancer patients, 44.0% had used two sources while 32.2% had used three or more sources for financing their cancer treatment. Most of the patients (78.0%) met their treatment cost by using multiple sources. A mere 5.8% of the patients used income as one of the sources for covering treatment cost, 48.6% used savings, 66.6% had resorted to loans & borrowings and 72.4% had either sold assets or borrowed to finance the cost of treatment.

**Table 4:** Sources of financing treatment and share to total cost of treatment

Source of financing	%	*n*	Mean amount spent from source (in ₹)	Average treatment cost (in ₹)	Source of financing as a share to total cost of treatment
Income	5.8	24	59,917	173,244	34.6
Savings	48.6	202	140,397	280,830	50.0
Selling assets, jewellery, property	11.8	49	251,939	357,209	70.5
Loans & borrowings	66.6	277	108,179	238,314	45.4
Contribution from friends	45.0	187	157,101	247,659	63.4
Insurance	39.7	165	106,536	202,673	52.6
Selling assets or borrowing (Distress financing)	72.4	301	195,195	247,384	78.9
1 Source	22.0	88	240,523	306,885	NA
2 + Sources	78.0	317	215,129	241,086	NA

NA: Not applicable

A set of logistic regression models were estimated to understand the significant predictors of distress financing (Table 5**).** The pairwise analysis (Model 1) indicates that CHE is a significant predictor of distress financing (OR:2.25; 95% CI:1.32, 3.86). The coefficient decreased marginally in Model 2 and 3 but remained statistically significant. In full model (Model 3), patients undergoing distress financing were almost two times more likely to be incurring CHE (OR:1.87, 95% CI: 1.01, 3.38). Other important predictors of distress financing are patients who had more than a year of treatment (OR: 2.21, 95% CI: 0.81, 6.06), and educated (OR: 2.53; 95% CI: 1.04, 6.16). The odds of distress financing were significantly lower among private patients (OR:0.36, 95% CI: 0.18, 0.72), who were from within Maharashtra (OR:0.52, 95% CI: 0.29,0.94) and who had any insurance (OR:0.45, 95% CI: 0.23, 0.87). Households with labour and self-employment as main source of income were two times more likely to incur distress financing than households with services as main source of income.

**Table 5:** Result of logistic regression analysis on significant predictors of distress financing

Distress financing	Model 1			Model 2			Model 3		
	OR	*p*-value	95% CI	OR	*p*-value	95% CI	OR	*p*-value	95% CI
**CHE**	**2.25**	0.003	[1.32, 3.86]	**1.89**	0.030	[1.06, 3.36]	**1.87**	0.048	[1.01, 3.38]
**Age**									
Up to 45 years ^®^				1.00			1.00		
Over 45 years				0.90	0.674	[0.56, 1.46]	1.04	0.868	[0.63, 1.74]
**Marital status**									
Not currently Married ^®^				1.00			1.00		
Currently Married				1.47	0.222	[0.79, 2.72]	1.38	0.327	[0.73, 2.61]
**Patients’ Type**									
General/Non-chargeable ^®^				1.00			1.00		
Private				**0.47**	0.020	[0.25, 0.89]	**0.36**	0.004	[0.18, 0.72]
**Stage of Cancer**									
Early Stage (I/II) ^®^				1.00			1.00		
Advanced Stage (III/IV)				0.89	0.617	[0.56, 1.42]	0.84	0.491	[0.52, 1.37]
**Type of treatment**									
All three treatment modalities (systemic therapy, radiotherapy and surgery)				1.19	0.564	[0.66, 2.13]	1.23	0.509	[0.67, 2.27]
At least one or two treatment modalities ^®^				1.00					
**Duration of Treatment (in months)**									
< 9 M ^®^				1.00			1.00		
9 M-12 M				0.98	0.940	[0.61, 1.59]	0.87	0.584	[0.53, 1.43]
> 12 M				2.60	0.054	[0.98, 6.89]	2.21	0.122	[0.81, 6.06]
**Patients Years of Schooling**									
Never Attended				**2.44**	0.024	[1.13, 5.30]	**2.27**	0.049	[1.00, 5.11]
Primary				1.82	0.213	[0.71, 4.65]	1.71	0.283	[0.64, 4.54]
Secondary				1.71	0.096	[0.91, 3.21]	1.82	0.084	[0.92, 3.59]
Higher Secondary				**2.41**	0.041	[1.04, 5.59]	**2.53**	0.041	[1.04, 6.16]
Higher Secondary and Above ^®^				1.00			1.00		
**Insurance**									
No insurance ^®^				1.00			1.00		
Any Insurance				**0.34**	< 0.001	[0.19, 0.61]	**0.45**	0.018	[0.23, 0.87]
**Household Size**									
1–4 members ^®^				1.00					
5 or more members				0.99	0.98	[0.64, 1.56]	0.96	0.878	[0.60, 1.55]
**Income Source**									
Agriculture							0.69	0.348	[0.32, 1.49]
Labour							**2.14**	0.026	[1.10, 4.18]
Self-Employed							**2.23**	0.025	[1.10, 4.52]
Service ^®^							1.00		
**Caste**									
General							0.82	0.456	[0.48, 1.39]
OBC							0.85	0.671	[0.41, 1.77]
SC/ST/Other ^®^							1.00		
**State**									
Within Maharashtra							**0.52**	0.031	[0.29, 0.94]
Outside Maharashtra ^®^							1.00		

## Discussion

Breast cancer treatment spans over a year and the estimates of financial catastrophe from cross sectional surveys are likely to be underestimated. Such estimates are desirable from longitudinal study design. In India, there is no comprehensive study that provides reliable estimates of financial catastrophe induced by breast cancer using longitudinal study design. This is the first ever study to estimate the extent of catastrophic health spending, impoverishment, and distress financing using data from a longitudinal cohort study in India. The study is unique because it used expenditure data collected on each visit of treatment from a tertiary hospital in Mumbai that provides subsidies and private treatment and had an adequate sample for disaggregated analyses.

The extent of financial catastrophe of breast cancer is very high in India as over 84.6% (95% CI: 80.8, 87.9%) households incurred CHE. Affected households in rural areas, belonging to poor economic condition, who came from outside of Maharashtra and households who primarily depend on agriculture for their livelihood are more likely to face CHE. Furthermore, over 70% of breast cancer patients benefitted from any type of reimbursement. However, the reimbursement (including health insurance) resulted in a mere 13.8% reduction in the CHE incidence. Moreover, most of the households with breast cancer patients relied on multiple sources to meet their treatment expenses. The patients who had to resort to borrowing and selling assets as a means of financing their treatment cost had been able to do so to the extent of about one-fifth of their total treatment cost. We found a strong association between distress financing and CHE. Distress financing was significantly higher among patients who had undergone treatment for over a year, had no education or had up to higher secondary education, and who relied on labour or self-employment as an income source. The multivariate analyses shows that the association remains statistically significant across the subsequent models after controlling for patient and household characteristics.

The estimates of CHE of breast cancer survivals are similar to those of China, Korea and other LMICs [18, 23]. A previous systematic review has shown that the pooled CHE for cancer is almost 70% in LMICs [15]. Our estimate is also comparable to CHE estimates of colorectal and cervical cancer in India [20, 22]. However, it was higher compared to NSS based estimates derived for any cancer [26, 35]. Our estimate of CHE is robust, as we have comprehensively recorded the cost of treatment in each visit of the patients to TMC over entire treatment period. The high CHE is also reflective of a high cost of treatment and lower reimbursement. Overall, screening for breast cancer is very low in India, thus the disease is detected at an advanced stage, pushing up the duration and the overall cost of treatment [2, 36]. The outcome of breast cancer worsens with delayed detection, thus increasing the overall cost of treatment, which makes households vulnerable to resorting to distress financing. The high cost of treatment and the consequent OOP payments after treatment leaves the household finances high and dry, and bereft of any savings or income forcing them to resort on distress financing. The indebtedness in fact increased during cancer treatment and possibly continued post treatment. Exposure to multiple sources of distress financing has pushed most households towards impoverishment as they incur high levels of CHE.

The financial protection provided by insurance and trusts reduces the bereavement caused by the high cost of breast cancer and reduce the incidence of CHE, and distress financing to some extent but it is not adequate. The increased financial burden of CHE is also possibly due to non-reimbursement of indirect costs such as accommodation, travel, and food of accompanying persons. Often, cancer is excluded from the list of diseases by health insurers or it needs specific insurance only for cancer which ostracizes those insured and uninsured from reaping the benefits, hindering them to come under the health insurance ambit. Reimbursement for diseases other than cancer is typically given only in case of hospitalization. However, cancer treatment mostly involves visits to out-patient departments (OPDs), and regular consultation with doctors, with few days of subsequent hospitalisation yet, reimbursement is not given for expensive doctor consultations and OPD visits.

Social health insurance coverage is a potent tool in tackling the burden of CHE and protecting some of the households with breast cancer patients. Provisions within the existing health insurance schemes, by increasing the scope, coverage, and compensation for cancer treatment can reduce financial catastrophe. Making cancer treatment free for all irrespective of the type of cancer would help to mitigate the burden of CHE among Indian households. There is a specific need of implementation and upgradation of the existing primary health centres (PHCs) for cancer screening and treatment. The Pradhan Mantri Jan Arogya Yojana (PM-JAY) scheme is a welcome move in this direction, which enhances the strength of the PHC’s and formation of new health and wellness centres (HWCs), which also need to implement cancer screening programmes. [37]

Patients diagnosed at an advanced stage of cancer are more likely to require expensive treatment, thus strategies for screening and early diagnosis need to be formulated [38]. The government and other stakeholders need to make a significant increase in health and health care investment to decrease the burden of CHE and ameliorate the financial stability of these households with breast cancer patients. Last, households below poverty line with cancer patients should be given long term financial help to recuperate from the downturns due to the disease.

There are some limitations of our study. First, the long-term financial consequences of breast cancer survivors cannot be analysed as we have not collected data after six months follow up. Second, the CHE in this study include direct medical and non-medical costs but not indirect costs (wage loss, time loss of patients and accompanying person). Thus, our estimates might be under estimated. Third, the societal costs related to a household with breast cancer may be very high, which in general is not reflected in the patients’ bills and the costs incurred by the accompaniments of the patients’ that are not reported in this study. Fourth, part of the data was collected during the COVID-19 pandemic which might potentially lead to longer treatment period, increased costs and discontinued treatment.

## Conclusion

The findings reveal that the incidence and intensity of CHE was high among households with breast cancer patients. Households resort to multiple sources and largely from borrowing, loan and selling assets for treatment. The strong and significant association between distress financing and CHE suggests that with an increase in CHE, distress financing also increases. The social and demographic factors do significantly affect distress financing. This study offers strong evidence using a comprehensive methodology that will guide in future to design multi-centre collaborative studies to comprehend the effects of CHE and distress financing of breast cancer treatment in a larger population that can be replicated elsewhere.

### Electronic supplementary material

Below is the link to the electronic supplementary material.

Supplementary Material 1

## Data Availability

All the authors involved in the study have access to the data collected as a part of this project. Sharing of the data with outside parties is at the discretion of Dr. Tabassum Wadasadawala, ACTREC, Navi Mumbai, Email: twadasadawala@actrec.gov.in and maybe considered on request.

## Abbreviations

TMC: Tata Memorial Centre
IIPS: International Institute for Population Sciences
CHE: Catastrophic Health Expenditure
OOP: Out of pocket
LMIC: Low-Middle Income Country
SDG: Sustainable Development Goals
HDI: Human Development Index
PM-JAY: Pradhan Mantri Jan Arogya Yojana
PHC: Primary Health Centre
HWC: Health and Wellness Centre
NSS: National Sample Survey
MPCE: Monthly per capita consumption expenditure
CTP: Capacity to pay
