# Supplementary material for: Catastrophic health expenditure and distress financing of breast cancer treatment in India: evidence from a longitudinal cohort study
Source: Int J Equity Health. 2024 Jul 23;23:145. doi: 10.1186/s12939-024-02215-2 (PMC11265332; doi:10.1186/s12939-024-02215-2)
Supplement: Supplementary file 1 — Supplementary Material 1 [file 12939_2024_2215_MOESM1_ESM.docx]

**SM Table 1: Consumption Schedule used in the survey**

| Sr. No. | 1. **USUAL EXPENDITURE IN LAST 30 DAYS** | Month after diagnosis of cancer (for patient and attendant)  (A) | Month after diagnosis  (for other HH members)  (B) | Month before diagnosis of cancer  (for all household member)  (C) |
| --- | --- | --- | --- | --- |
| 18.1 | Consumption on Food items (staple food, grocery, oil, vegetables, fruits, dairy, oil, egg, meat, etc.) |  |  |  |
| 18.2 | Expenditure on Utility bills (mobile, electricity, water, cooking fuel etc.) |  |  |  |
| 18.3 | Expenditure on Travel (car fuel or public transport) |  |  |  |
| 18.4 | Expenditure on Entertainment (recreation, hobbies, sports) |  |  |  |
| 18.5 | Habits (smoking, alcohol etc..) |  |  |  |
| 18.6 | Consumer services (maid, cook, laundry, car wash, newspaper) |  |  |  |
| 18.6.1 | Rentals (house only) |  |  |  |
| 18.6.2 | Total monthly expenditure |  |  |  |
| 18.6.3 | If yes have received subsidized food, accommodation or any other facility, please give equivalent amount of subsidized facility |  |  |  |
| 18.6.4 | Total monthly usual expenditure |  | | |
|  | 1. **USUAL EXPENDITURE IN LAST ONE YEAR** |  | | |
| 18.7 | Education (books, fees) in last one year |  | | |
| 18.8 | Clothes in last one year |  | | |
| 18.9 | Insurance premium (life) in last one year |  | | |
| 18.10 | Insurance premiums (health) in last one year |  | | |
| 18.11 | Total usual expenditure in last one year |  | | |
|  | 1. **NON-USUAL EXPENDITURE IN LAST ONE YEAR** |  | |  |
| 18.12 | Others (non-usual expenditure in last one month) |  |  |  |
